# Pediatric Robotic Surgery in Romania: Review of the First 71 Cases Using the da Vinci Platform

**DOI:** 10.3390/children13060738

**Published:** 2026-05-26

**Authors:** Vlad-Laurentiu David, Maria-Corina Stanciulescu, Emil-Radu Iacob, Calin-Marius Popoiu

**Affiliations:** 1 Department of Pediatric Surgery and Orthopedics, “Victor Babes” University of Medicine and Pharmacy, 300041 Timisoara, Romania; david.vlad@umft.ro (V.-L.D.); radueiacob@umft.ro (E.-R.I.); mcpopoiu@umft.ro (C.-M.P.); 2 Department of Pediatric Surgery and Orthopedics, “Louis Turcanu” Emergency Children’s Hospital, 300011 Timisoara, Romania

**Keywords:** pediatric robotic surgery, da Vinci XI surgical system, initial experience, minimally invasive surgery, learning curve, Romania

## Abstract

Background: Robotic-assisted surgery has increasingly been adopted in pediatric surgical practice; however, data from early implementation stages remain limited. Materials and methods: We conducted a prospective audit of the first 71 pediatric robotic-assisted procedures performed over a 24-month period using the da Vinci Xi platform in a tertiary pediatric center. Patient characteristics, surgical indications, perioperative parameters, and postoperative outcomes were analyzed. Results: A total of 71 procedures were performed in 71 patients (39 girls, 32 boys; mean age 4.46 years). The most frequent procedures were cholecystectomy (n = 19), ovarian tumor excision (n = 14), and pyeloplasty (n = 13). Mean operative time was 90 ± 65.30 min. Intraoperative complications occurred in 9.9% of cases, conversion to open surgery in 2.8%, and postoperative complications in 2.8%. Trocar insertion time and docking time improved significantly during the second year (*p* < 0.05). No mortality or long-term complications were recorded. Conclusions: Robotic-assisted pediatric surgery is feasible and safe, with acceptable complication rates and favorable early outcomes. Progressive improvement in operative setup parameters reflects a measurable learning curve.

## 1. Introduction

Minimally invasive surgery (MIS) has become a cornerstone of contemporary pediatric surgical practice, supported by substantial evidence demonstrating reduced postoperative pain, shorter hospitalization, faster recovery, and improved cosmetic outcomes compared with open surgery [1]. Conventional laparoscopy is now widely established across a range of pediatric procedures, including in neonates and infants, where its safety and efficacy have been consistently demonstrated [2,3]. Consequently, it represents the current standard approach for many surgical indications in children.

Despite these advantages, conventional laparoscopy is associated with several technical limitations, including two-dimensional visualization, restricted instrument mobility, amplification of physiological tremor, and a demanding learning curve for advanced skills such as intracorporeal suturing. These constraints are particularly relevant in pediatric surgery, where limited working space and small anatomical structures necessitate a high level of precision and control.

Robotic-assisted surgery has been developed as an advanced MIS modality to address these limitations. Robotic platforms provide three-dimensional high-definition visualization, tremor filtration, motion scaling, and articulated instruments with enhanced degrees of freedom [4]. In addition, ergonomic advantages for the surgeon may contribute to more consistent operative performance. These features are especially advantageous in pediatric procedures requiring fine dissection and complex reconstructive techniques within confined anatomical spaces. However, direct comparison with conventional laparoscopy is beyond the scope of the present study.

Since its initial adoption in pediatric urology, robotic-assisted surgery has expanded to other pediatric subspecialties, including general surgery, thoracic surgery, and gynecology [5,6]. Existing studies have demonstrated the feasibility and safety of robotic approaches in children, with perioperative outcomes comparable to those of conventional laparoscopy in selected cases [4,5,6]. However, evidence regarding clear clinical superiority remains limited, and further data are required to define its role across different indications.

The implementation of robotic surgery in pediatric practice remains heterogeneous. Key influencing factors include system acquisition and maintenance costs, availability of appropriately scaled instrumentation, institutional infrastructure, surgeon training, and procedural volume. From an applied perspective, the integration of robotic systems into pediatric surgical workflows represents a complex process requiring adaptation at both technical and organizational levels.

In this context, early institutional experiences are particularly valuable, as they provide practical insights into program implementation, including patient selection, operative setup, workflow optimization, and the learning curve associated with robotic techniques. Furthermore, such reports contribute to the evaluation of perioperative outcomes and the identification of potential limitations during the initial adoption phase [7,8,9]. Despite increasing interest, data from centers in the early stages of robotic program development remain limited.

The aim of this study is to present our initial experience with robotic-assisted pediatric surgery at a single tertiary care center. We analyze patient characteristics, surgical indications, operative parameters, and perioperative outcomes to assess feasibility, safety, and early results. This study is descriptive in nature and does not aim to compare robotic-assisted surgery with conventional laparoscopic approaches. By focusing on the implementation phase, this study seeks to provide clinically relevant and technically applicable insights that may support other institutions in the adoption and integration of robotic-assisted techniques in pediatric surgery.

## 2. Materials and Methods

We conducted an audit of the first two years of using the robotic da Vinci Xi platform in our institution. Our center is a tertiary pediatric hospital covering the full spectrum of pediatric surgery, including general, digestive, neonatal, urological, gynecological, and thoracic procedures.

The robotic platform was acquired in December 2017. A team consisting of two console surgeons and two assistant surgeons completed the training program provided by Intuitive Surgical and were certified to use the da Vinci system. The platform used was the da Vinci Xi with four arms.

The system offers an extended range of instruments for minimally invasive surgery, including dissecting and grasping forceps, scissors, monopolar hook, bipolar and monopolar energy devices, vessel sealers, and stapling devices. However, only 8 mm instruments were available. These instruments are fully articulated, offering seven degrees of intracorporeal freedom. The optical system was also 8 mm and provided three-dimensional stereoscopic vision.

The first robotic-assisted pediatric procedure in Romania was performed on 9 February 2018. Over a 24-month period, 71 robot-assisted procedures were performed in children.

Patient selection for the robotic surgery program was based on pathological condition (suitability for minimally invasive surgery), anatomical feasibility (adequate working space, weight over 10 kg and at least 8 cm distance available between trocar placement sites on the abdominal wall), comorbidities, and overall clinical condition (no contraindication for minimal invasive surgery). Consecutive patients that met the criteria were included in the program. During the early implementation phase, a cautious and progressive approach was adopted, with initial preference for cases of moderate complexity to facilitate team familiarization with the platform. As experience increased, more complex cases were gradually included. The study was designed as a prospective audit, with predefined parameters, timing, and methodology.

We have previously reported the financial aspects of implementing a robotic surgery program [10]. In this study, we focused on clinical characteristics. For each patient, we recorded demographic and medical data, including age, sex, diagnosis, comorbidities, surgical procedure, medical treatment, reintervention rate, intraoperative and postoperative complications, and length of hospital stay. Complications were classified according to the Clavien–Dindo classification system [11].

Perioperative data included preoperative time, trocar insertion time, docking time, number of robotic arms used, use of accessory ports, intraoperative complications, and conversion rate.

- Preoperative time (PT): from patient entry into the operating room to skin incision
- Trocar insertion time (TI): time required to create pneumoperitoneum and place trocars
- Docking time (DT): time required to position the robotic cart and connect the arms
- Operative time (OT): from docking to skin closure
- Total operating room time (TOR): total time spent in the operating room

Statistical analysis was performed using IBM SPSS Statistics (version 23). The statistical approach was primarily descriptive and exploratory. Normality was assessed using the Shapiro–Wilk test. As none of the variables followed a normal distribution, data were reported as range, median, standard deviation (SD), and percentages. Comparisons were performed using the Mann–Whitney U test. A *p*-value < 0.05 was considered statistically significant.

## 3. Results

The cohort included 39 girls and 32 boys, aged 1.3–19 years (mean 4.46 years). Weight ranged from 10 to 118 kg (mean 49.00 ± 25.56 kg), and height from 75 to 182 cm (mean 160.00 ± 23.45 cm).

Of the 71 procedures, 40 were performed in the first year and 31 in the second year. The treated conditions and procedures are presented in Table 1.

All procedures were performed under general anesthesia with orotracheal intubation. Patient positioning, table inclination, and trocar placement varied depending on the surgical site.

- PT: 15–100 min (mean 45 ± 18.16)
- TI: 5–30 min (mean 15 ± 5.31)
- DT: 5–30 min (mean 10 ± 4.81)
- OT: 30–335 min (mean 90 ± 65.30)
- TOR: 85–420 min (mean 165 ± 72.35)

Compared with the first year, TI (*p* = 0.035) and DT (*p* = 0.010) were significantly shorter in the second year, while PT, OT, and TOR remained similar.

Intraoperative complications occurred in 7 patients (9.9%). Conversion to open surgery was required in 2 cases (2.8%), and reintervention in 5 cases (7.1%). Postoperative complications occurred in 2 patients (2.8%), including one who suffered a previous intraoperative complication requiring conversion. All of the intraoperative complications, postoperative complications and reinterventions (Table 2) happened in the first year (*p* = 0.00). Conversion rate was similar in the two years comprised in this report, one for each one, including one splenectomy due to bleeding from a branch of the splenic artery and one additional case of adrenalectomy due to technical difficulties.

Overall outcomes were favorable for all patients, with no mortality and no long-term complications or sequelae.

## 4. Discussion

This study presents our institutional experience with the first 71 consecutive robotic-assisted pediatric procedures, representing not only the initial implementation of robotic surgery in our center but also the first reported series of its kind in Romania. As such, it provides important real-world evidence on the feasibility, safety, and practical integration of robotic technology into pediatric surgical practice in an emerging setting. Beyond its descriptive value, this study contributes to bridging a significant geographic and institutional gap in the current literature, where data from early-stage robotic programs remain limited [7,8,9].

The wide spectrum of pathologies addressed, including digestive, urological, gynecological, and general surgical conditions, highlights the versatility and adaptability of the robotic platform in pediatric surgery. This diversity is consistent with reports demonstrating the applicability of robotic systems across pediatric subspecialties, particularly in reconstructive urology and complex abdominal surgery [12,13,14]. Importantly, our deliberate inclusion of procedures with varying levels of complexity reflects a pragmatic and scalable implementation strategy. This approach not only allowed for comprehensive evaluation of the system across multiple clinical scenarios but also facilitated structured skill acquisition within the surgical team. Such findings reinforce the concept that successful adoption of robotic surgery is not procedure-specific but program-dependent, relying heavily on systematic training and progressive case selection [12]. The selection criteria were very loose, meaning that basically we excluded only the patient that had contraindications for minimal invasive surgery and the ones that were feasible from an anatomical point of view. The system has a technical limitation with the exclusive use of 8 mm instruments. In smaller pediatric patients, instrument size may restrict maneuverability and limit applicability in confined anatomical spaces. This constraint may have influenced case selection, particularly in younger or lower-weight patients. The inclusion of a broad spectrum of procedures introduces significant heterogeneity in operative complexity, duration, and risk profile. While this reflects real-world implementation, it limits procedure-specific conclusions and complicates interpretation of aggregated outcomes. Future studies focusing on homogeneous procedure groups will be necessary to better define the role of robotic assistance in specific indications.

Although some procedures performed in this series, such as appendectomy or inguinal hernia repair, may not fully exploit the technological advantages of robotic systems, their inclusion should not be underestimated. These procedures play a critical role in the early phase of implementation by enabling workflow standardization, improving docking efficiency, and enhancing team coordination. From a translational perspective, this stepwise approach may represent a reproducible model for other centers aiming to safely introduce robotic platforms into pediatric practice [7,8,12].

Conversely, the predominance of cholecystectomy in our series reflects its role as both a clinically relevant and technically accessible procedure during the learning curve. In our experience, robotic cholecystectomy provided stable visualization and adequate instrument articulation, but current evidence does not demonstrate clear clinical superiority over laparoscopy in pediatric populations [15]. This underscores a critical point: the value of robotic surgery should not be universally assumed but rather selectively applied, with clear consideration of indication, complexity, and added benefit.

The procedural distribution observed in our cohort, with cholecystectomy, ovarian tumor excision, and pyeloplasty being the most frequent, is consistent with existing literature. Notably, robotic pyeloplasty remains one of the most widely accepted indications in pediatric robotic surgery and is often considered a benchmark procedure [16]. In our experience, the robotic approach provided excellent surgical control and reproducibility, particularly in reconstructive steps requiring precise intracorporeal suturing. These findings are consistent with existing literature suggesting that robotic assistance may offer tangible technical advantages in complex reconstructive procedures, potentially translating into improved functional outcomes [8,17].

Our experience with gynecological and splenic procedures, although limited, further supports the feasibility of robotic surgery across less commonly reported pediatric indications. The ability to perform precise dissection in confined anatomical spaces, such as the pelvis, represents a clear technical advantage. However, the occurrence of a major complication requiring conversion during splenic surgery highlights the ongoing importance of careful selection of the patients and underscores that robotic technology does not eliminate the inherent challenges of complex surgical anatomy [18].

The overall complication rate in our series (12.7%) falls within the range reported in the literature (5–15%) [5,12]. Importantly, all complications occurred during the first year, providing strong evidence of a measurable and clinically relevant learning curve. The absence of complications in the second year, combined with the significant reduction in trocar insertion and docking times, suggests not only improved technical proficiency but also enhanced team performance and system integration. These findings emphasize that the learning curve in robotic surgery is multifactorial, encompassing not only the console surgeon but the entire operative team and workflow.

Although the surgical team completed formal training and certification, ongoing quality assurance measures, structured performance evaluation, and peer-review mechanisms were not formally analyzed in this study. These elements are important components of sustainable robotic program development. Learning curve analyses suggest that approximately 15–25 cases are required for stabilization of operative times in robotic pyeloplasty [5,19]. In our series, only 13 pyeloplasty procedures were performed, and therefore this threshold was not reached, limiting procedure-specific learning curve assessment. The relatively short docking times and the improvement of these parameters (TI&DT), in the second year of implementation in our series, indicate increasing team efficiency, consistent with institutional maturation of robotic programs. Importantly, operative duration in our study is likely to reflect procedural heterogeneity rather than platform inefficiency. This study focuses primarily on perioperative outcomes. Functional and long-term results were not systematically evaluated. For example, outcomes such as resolution of obstruction following pyeloplasty, recurrence rates for ovarian tumors, or long-term organ function were not included. These parameters are essential for assessing true clinical efficacy and should be addressed in future longitudinal studies.

The conversion rate (2.8%) observed in this study is consistent with published benchmarks (0–5%) [5,12] and further supports the safety of robotic-assisted approaches. Notably, conversions were associated with anatomical complexity and early-phase decision-making, highlighting the importance of experience in patient selection and intraoperative judgment. As institutional expertise increases, these factors are expected to improve, further optimizing outcomes.

Postoperative morbidity was low (2.8%), with no mortality or long-term complications observed, findings that are consistent with previously published data [5,12]. These results reinforce the favorable safety profile of robotic-assisted pediatric surgery and support its integration into clinical practice under appropriate conditions. Importantly, our findings suggest that the introduction of robotic surgery does not compromise patient safety during the early implementation phase when conducted within a structured and supervised framework.

From an applied sciences perspective, this study provides valuable insight into the real-world challenges of integrating advanced surgical technologies into clinical practice. The observed improvements in efficiency over time demonstrate that optimization of robotic surgery extends beyond technical execution to include workflow standardization, team dynamics, and institutional adaptation. These aspects are critical for maximizing the benefits of robotic systems and should be considered integral components of program development.

Although cost is recognized as a major limitation of robotic surgery, no formal economic analysis was performed in this study. While financial aspects have been previously reported by our group [10], the absence of integrated cost-effectiveness data limits conclusions regarding the economic impact of robotic program implementation. The high initial investment, ongoing maintenance costs, and consumable expenses pose substantial challenges, particularly in pediatric settings with lower procedural volumes [10]. While improved precision and potential reductions in complication rates may offer indirect economic benefits, robust cost-effectiveness analyses are still lacking. Addressing this gap represents an important direction for future research. Future research should focus on prospective, multicenter studies with standardized protocols and comparative designs to better define the indications, benefits, and economic impact of robotic-assisted surgery in pediatric populations. In addition, further investigation into learning curve metrics and outcome optimization strategies will be essential to fully realize the potential of robotic technology in this field.

This study has several limitations, including its single-center design, relatively small sample size, and procedural heterogeneity. Also, the statistical analysis was primarily descriptive, without multivariate modeling, long-term and functional outcomes were not assessed, and no formal cost analysis was included. The absence of a control group limits direct comparison with laparoscopic or open approaches, and the variability in case complexity may influence outcome interpretation, meaning that the findings should be interpreted as purely descriptive of our institutional experience. Nevertheless, the strength of this study lies in its consecutive case series design and its focus on the early implementation phase, providing practical insights that are highly relevant for other institutions. However, these findings should be interpreted in the context of a single-center experience, limited sample size, procedural heterogeneity, and absence of long-term follow-up.

## 5. Conclusions

Compared with published pediatric robotic series, our results demonstrate comparable operative times, low conversion rates (2.8%), acceptable complication rates, and excellent overall outcomes, with no mortality or long-term sequelae.

These findings support the conclusion that robotic-assisted surgery in children is a safe and effective minimally invasive approach across a wide range of indications, provided that appropriate patient selection and institutional expertise are ensured. Further prospective, comparative, and multicenter studies with standardized protocols and long-term follow-up are required to better define the clinical role and cost-effectiveness of robotic-assisted surgery in pediatric populations.

## Figures and Tables

**Table 1:** Treated conditions and performed procedures.

No.	Condition	Procedure	No.
1	Gallbladder lithiasis	Cholecystectomy	19
2	Ovarian tumor	Tumor excision	14
3	Ureteropelvic junction obstruction	Pyeloplasty	13
4	Varicocele	Palomo procedure	9
5	Appendicitis	Appendectomy	4
6	Hereditary spherocytosis	Splenectomy	3
7	Inguinal hernia	Hernia repair	2
8	Splenic cyst	Partial splenectomy	2
9	Dysplastic kidney	Nephrectomy	2
10	Adrenal adenoma	Adrenalectomy	1
11	Morgagni hernia	Hernia repair	1
12	Intestinal duplication cyst	Cyst excision	1
	Total		71

**Table 2:** Intraoperative and postoperative complications and solutions.

Procedure	Complication/Incident	Management	No. of Cases	Clavien–Dindo
Cholecystectomy	Gallbladder perforation with spillage	Aspiration, lavage, abdominal drainage, antibiotics	3	I
Cholecystectomy	Duodenal perforation	Suturing, antibiotics	1	IIIb
Cholecystectomy	Biliary fistula	Reintervention, Kehr tube placement	1	IIIb
Pyeloplasty	Failure of antegrade JJ stent placement intraoperatively	Retrograde cystoscopic insertion 1 day postoperatively	1	IIIb
Pyeloplasty	Failure of antegrade JJ stent placement intraoperatively	Nephrostomy; cystoscopy and ureterovesical reimplantation for associated ureterovesical stenosis	1	IIIb
Splenectomy	Bleeding from a branch of the splenic artery	Conversion to open surgery	1 *	IIIb
	Surgical wound seroma	Surgical drainage	1 *	II

* Both complications occurred in the same patient.

## Data Availability

The data presented in this study are available upon request from the corresponding author (M.C.S.).

## Abbreviations

The following abbreviations are used in this manuscript:

DT	Docking time
PT	Trocar insertion time
SD	Standard deviation
TI	Linear dichroism
TOR	Total operating room time
